# Effects of the Phytochemical Combination PB123 on Nrf2 Activation, Gene Expression, and the Cholesterol Pathway in HepG2 Cells

**DOI:** 10.21926/obm.icm.2201002

**Published:** 2022-01-10

**Authors:** Brooks M. Hybertson, Bifeng Gao, Joe M. McCord

**Affiliations:** 1. Pathways Bioscience, Aurora, CO 80045, USA;; 2. Department of Medicine, Division of Pulmonary Sciences and Critical Care Medicine, University of Colorado Anschutz Medical Campus, Aurora, CO 80045, USA

**Keywords:** Nrf2, KEAP1, oxidative stress, aging, cholesterol, nutrition

## Abstract

There has been a long history of human usage of the biologically-active phytochemicals in *Salvia rosmarinus*, *Zingiber officinale*, and *Sophora japonica* for health purposes, and we recently reported on a combination of those plant materials as the PB123 dietary supplement. In the present work we extended those studies to evaluate activation of the nuclear factor erythroid 2-related factor 2 (Nrf2) transcription factor and differential gene expression in cultured HepG2 (hepatocellular carcinoma) cells treated with PB123. We determined transcriptome changes using mRNA-seq methods, and analyzed the affected pathways using Ingenuity Pathway Analysis and BioJupies, indicating that primary effects included increasing the Nrf2 pathway and decreasing the cholesterol biosynthesis pathway. Pretreatment of cultured HepG2 cells with PB123 upregulated Nrf2-dependent cytoprotective genes and increased cellular defenses against cumene hydroperoxide-induced oxidative stress. In contrast, pretreatment of cultured HepG2 cells with PB123 downregulated cholesterol biosynthesis genes and decreased cellular cholesterol levels. These findings support the possible beneficial effects of PB123 as a healthspan-promoting dietary supplement.

## 1. Introduction

Aging and age-related diminishment of the body’s antioxidant defenses are associated with a variety of disorders and diseases [1–5]. Dietary composition has significant influence on the body’s ability to fight against oxidative stress, which is one of the ways that dietary intake plays a role in healthy aging [6, 7]. In the past this was largely attributed to direct scavenging of oxidants by compounds consumed in the diet [8–10], but in recent years the focus has shifted to endogenous protection mechanisms and to understanding the health benefits of dietary components based on their ability to activate endogenous defenses, for example by inducing the increased expression of antioxidant enzyme genes [11–14].

Nuclear factor erythroid 2-related factor 2 (Nrf2) is a transcription factor that regulates the gene expression of a wide variety of cytoprotective phase II detoxification enzymes and antioxidant enzymes by engaging the antioxidant-responsive element (ARE) found in the promoter regions of these genes. Based on extensive prior work, the ARE is a promoter element that regulates the expression of many antioxidant, anti-inflammatory, and cytoprotective genes [15–23].

Aging is associated with diminishment of cytoprotective Nrf2 [24–27], which means that aging-related stressors coincide with a decreased ability to protect against them [27, 28]. Lower nuclear Nrf2 levels have been observed with advanced age in both rats and people [24, 27], along with decreased antioxidant defense and repair capabilities [27]. Naturally long-lived species like the naked mole-rat have been shown to have markedly elevated levels of Nrf2 activation [29]. Total and LDL cholesterol levels tend to increase with age, and concomitantly the risk for atherosclerosis and coronary heart disease also tends to increase with age [30, 31]. Notably, previous investigations have indicated that dietary supplementation with ginger, a Nrf2 activator, correlated with decreased cholesterol levels in human subjects [32].

In the present work, we examine effects of the PB123 dietary supplement, a combination of phytochemical compounds from *Salvia rosmarinus*, *Zingiber officinale*, and the bioflavonoid luteolin [33–51]. Rosemary (*Salvia rosmarinus*) has been utilized against a variety of health issues [1], based on reported anti-inflammatory [52], antioxidant [49–51], and antimicrobial benefits [53, 54]. Ginger (*Zingiber officinale*) is a member of the Zingiberaceae family of plants with over 2500 years of recorded use in traditional therapies, commonly with emphasis on anti-inflammatory, analgesic, and digestive system benefits [55–59]. Luteolin (found in many food and vegetable sources) [60–64] has frequently been used as a dietary supplement based on reported antioxidant [47], neurological [45], and anti-inflammatory benefits [44, 60, 65].

In our prior work, Nrf2-dependent genes were shown to be upregulated in alveolar epithelial cells isolated from HIV-1 transgenic rats after dietary administration of PB123 [66]. In the present work, we studied the ability of the PB123 combination to activate the Nrf2 pathway and upregulate antioxidant, anti-inflammatory, and other cell protective genes in HepG2 cells, and we determined its protection of cultured HepG2 cells against oxidative stress and dyslipidemia.

## 2. Materials and Methods

### 2.1 Materials and Reagents

Plant extracts: ginger root extract from *Zingiber officinalis* (standardized to 20% gingerols) and rosemary extract from *Salvia rosmarinus* (standardized to 6% carnosol; 15% carnosic acid) were obtained from Flavex (Rehlingen, Germany); luteolin (from *Sophora japonica*, standardized to 98% luteolin) was obtained from Jiaherb (Pine Brook, NJ, USA). Solvent extracts of PB123 powder were prepared by mixing a 10:5:1 mass ratio of rosemary, ginger, and luteolin powders then extracting the mixed powder (50 mg/mL) overnight in ethanol and collecting the supernatant [66, 67]. Cell culture: antibiotics and culture media powder were obtained from Thermo Fisher Scientific (Waltham, MA, USA). Reagents and assays: The intracellular lipid staining assay (Steatosis Colorimetric Assay Kit), ERK1/2 inhibitor (PD98059, CAS 167869-21-8), and Nrf2 inhibitor (AEM1, CAS 1030123-90-0) were obtained from Cayman Chemical (Ann Arbor, MI, USA). The cholesterol assay (Cholesterol/Cholesterol Ester-Glo) was obtained from Promega (Promega Corporation, Madison, WI, USA). Cumene hydroperoxide (CAS 80-15-9) and all other reagents were obtained from Sigma-Aldrich (St. Louis, MO, USA).

### 2.2 Cell Culture

For the genomic, lipid, and cytoprotection experiments we used the human hepatocellular carcinoma HepG2 cell line. For Nrf2 activation experiments we used HepG2 cells that had been stably transduced with a Nrf2-dependent firefly luciferase gene construct (HepG2-ARE cells), kindly provided by Dr. S.O. Simmons [68]. HepG2 cells are appropriate for the present work because they exhibit normal Nrf2 activation properties [69], lack Nrf2/KEAP1 mutations, and have previously been shown to be suitable for metabolic studies [70]. The HepG2 and HepG2-ARE cells were cultured and maintained by standard methods as previously described [67]. Cell viability was determined using a cell counting kit-8 (CCK8) assay (Dojindo Molecular Technologies, Inc., Rockville, MD, USA). Briefly, cells were assayed for viability by adding CCK8 solution, incubating at 37 °C, and measuring absorbance at 450 nm using a microplate spectrophotometer (Bio-Tek, Winooski, VT, USA). Absorbance values were normalized to the readings from vehicle control cells, and the data was presented as viable cells percentage relative to vehicle control cells.

### 2.3 Nrf2 Reporter Gene Assays

The HepG2-ARE promoter/reporter cells were used for assaying Nrf2 activation, measured as relative light units (RLU) as previously described [67]. For synergy experiments, cells were treated with combinations of rosemary, ginger, and luteolin extracts and with the corresponding concentrations of extracts of each individual agent. PB123 synergy was visualized by comparing the Nrf2 activation signal from the PB123 combination with the sum of the signals induced by treatments with the individual ingredient extracts with a range of concentrations of PB123 extract (1.6, 3.2, 4.8, 6.4, 8 μg/mL) or with the corresponding amounts of rosemary extract (1, 2, 3, 4, 5 μg/mL), ginger extract (0.5, 1, 1,5, 2, 2.5 μg/mL), and luteolin (0.1, 0.2, 0.3, 0.4, 0.5 μg/mL) alone. Combinatorial Nrf2 activation calculations using synergy reference models and data from a checkerboard style layout of combinations of rosemary extract (0–24 μg/mL) and ginger extract (0–12 μg/mL) were performed using calculation tools at synergyfinder.org.

### 2.4 Gene Expression Assays

#### 2.4.1 Cell Culture and RNA Isolation

HepG2 cells were treated for 16h in 24-well plates with 0 (vehicle control) or 12 μg/mL PB123 (as an extract of 50 mg/mL in 100% ethanol), with 4 biological replicates per treatment group. The cell culture and RNA isolation was performed as previously described [67]. Briefly, after the treatment period the total RNA was isolated from the cells using Trizol, then purified using Qiagen RNeasy clean-up columns (Qiagen Inc., Valencia, CA, USA). The concentration of RNA in each sample was measured using absorbance at 260 nm (A260) with a NanoDrop spectrophotometer (Thermo Fisher Scientific, Waltham, MA, USA). The RNA integrity in the samples was determined using Agilent TapeStation 4200 (Agilent, Santa Clara, CA, USA) at the University of Colorado Genomics and Microarray Core facility.

#### 2.4.2 mRNA-seq Assays

##### mRNA-seq Library Preparation.

Samples containing 10–100 ng of total RNA were used to prepare the Illumina NGS libraries according to manufacturer’s instructions for the NuGEN Universal Plus mRNA-Seq (Tecan Genomics, Redwood City, CA, USA). In this method, polyA selection is used to isolate mRNA from total RNA, which is then fragmented and primed for creation of double-stranded cDNA fragments, which are then amplified, size-selected, and purified for cluster generation.

##### Sequencing.

The mRNA template libraries were then sequenced as paired end 150 bp reads on the Illumina NovaSeq 6000 (Illumina, San Diego, CA, USA) at the University of Colorado Genomics and Microarray Core facility (Aurora, CO, USA). We sequenced at a depth that provides ~40M 2X150 bases reads per sample.

##### mRNA-seq Profiling.

The sequencing data was processed for differential gene expression as previously described [67]. The computational pipeline for analyzing the derived sequences utilized GSNAP [71], Cufflinks [72], and R for sequence alignment and determination of differential gene expression [73]. In short, GSNAP was used to map the generated reads to the human genome (GRCH38) [71], Cufflinks was used to derived expression (FPKM) [72], and R was used to analyze differential gene expression with ANOVA, with a false discovery rate (FDR) < 0.05 as the cutoff. The transcriptomic data was examined by pathway analysis using Ingenuity Pathway Analysis (Qiagen, Germantown, MD, USA). For further profiling, the raw sequencing data was also processed using Biojupies ([74, 75]) to identify which pathways of interest were modified by PB123.

### 2.5 Protein Assays

The Human HMOX1 PicoKine ELISA Kit (Boster Biological Technology, Pleasanton, CA, USA) was used to determine heme oxygenase-1 (HMOX1) protein levels in HepG2 cell lysates according to the manufacturer’s instructions as previously described [67]. Total protein in the lysates was measured using the method of Lowry [76].

### 2.6 Total Cholesterol Assays

The total cholesterol level in cultured HepG2 cells was measured using a chemiluminescent enzymatic assay kit according to manufacturer’s instructions (Cholesterol/Cholesterol Ester-Glo^™^ Assay kit, Promega, Madison, WI). Briefly, after 24 hr of treatment with PB123, the medium was removed and the HepG2 cells were washed 2x with PBS then lysed using the Cholesterol Lysis Solution. Cell lysate aliquots (50 μl) were loaded onto a white, opaque 96-well plate along with cholesterol standards of known concentration, 50 μL of Cholesterol Detection Reagent with Esterase enzyme reagent was added to each well and incubated at room temperature for 1 hour, then luminescence from each well was measured using a platereader luminometer.

### 2.7 Intracellular Lipid Assays

The intracellular lipid level in cultured HepG2 cells was measured using an Oil Red O stain based assay kit according to manufacturer’s instructions (Steatosis Colorimetric Assay Kit, Cayman Chemical, Ann Arbor, MI). Briefly, HepG2 cells were treated for 24 h with 0, 5, or 12 μg/mL PB123, then stained with Oil Red O, lysed, and dye extracted using solutions in the Steatosis Colorimetric Assay Kit, followed by measurement of the extracted Oil Red O dye by absorbance at 492 nm using the platereader to determine relative intracellular lipid level.

### 2.8 Cytoprotection Assays

To evaluate protective effects against oxidative stress, HepG2 cells were pretreated with PB123, with or without ERK1/2 inhibition by PD98059, and then challenged with cumene hydroperoxide (CH) as previously described [67]. Cytotoxic effects caused by CH were determined by measuring cell viability using CCK8 assay as described above.

### 2.9 Statistical Analysis

Data are presented as the mean ± SEM (standard error of the mean) of multiple replicates. Significance of observed differences in the means were evaluated by one-way ANOVA and Tukey’s multiple comparisons testing or by Student’s *t* test for unpaired data using Prism 9 software (GraphPad Software, version 9.3.0, San Diego, CA, USA). A *p* value < 0.05 was considered statistically significant.

## 3. Results

### 3.1 Nrf2 Activation and Synergy

#### 3.1.1 Nrf2 Activation

**Figure 1:**
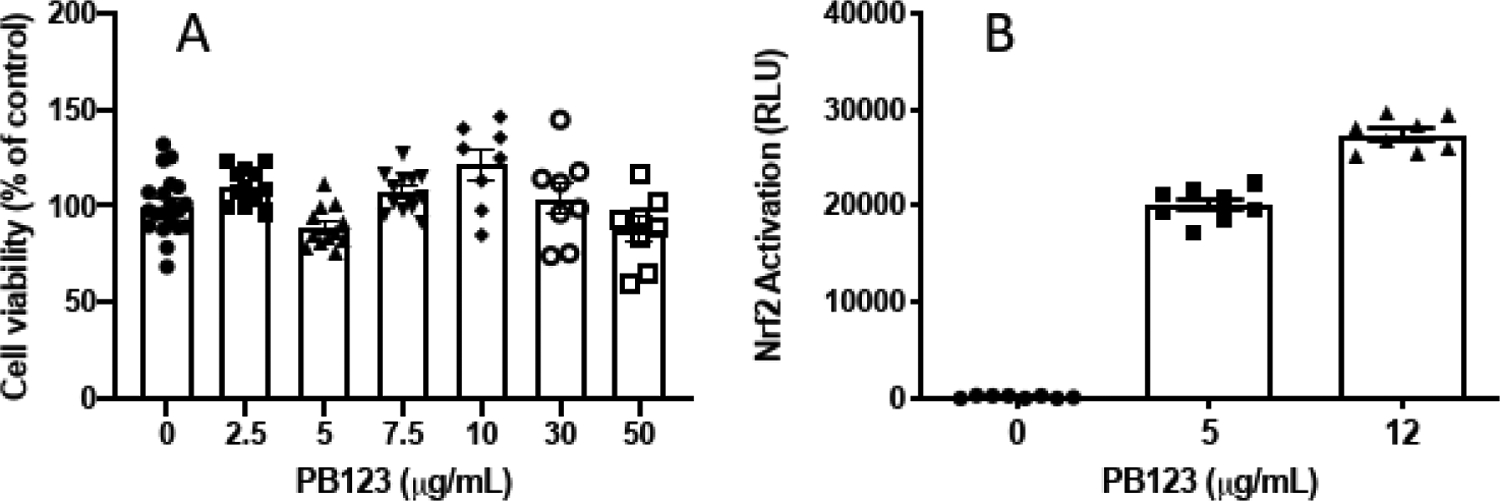
Effect of PB123 treatment on HepG2 cells. (A) PB123 was not toxic to HepG2 cells, measured by treating the cells for 24h with PB123 then determining cell viability by CCK8 assay. (B) PB123 activated Nrf2 in HepG2-ARE cells in a dose-dependent manner (p<0.05) by 5 and 12 μg/mL PB123.

**Figure 2:**
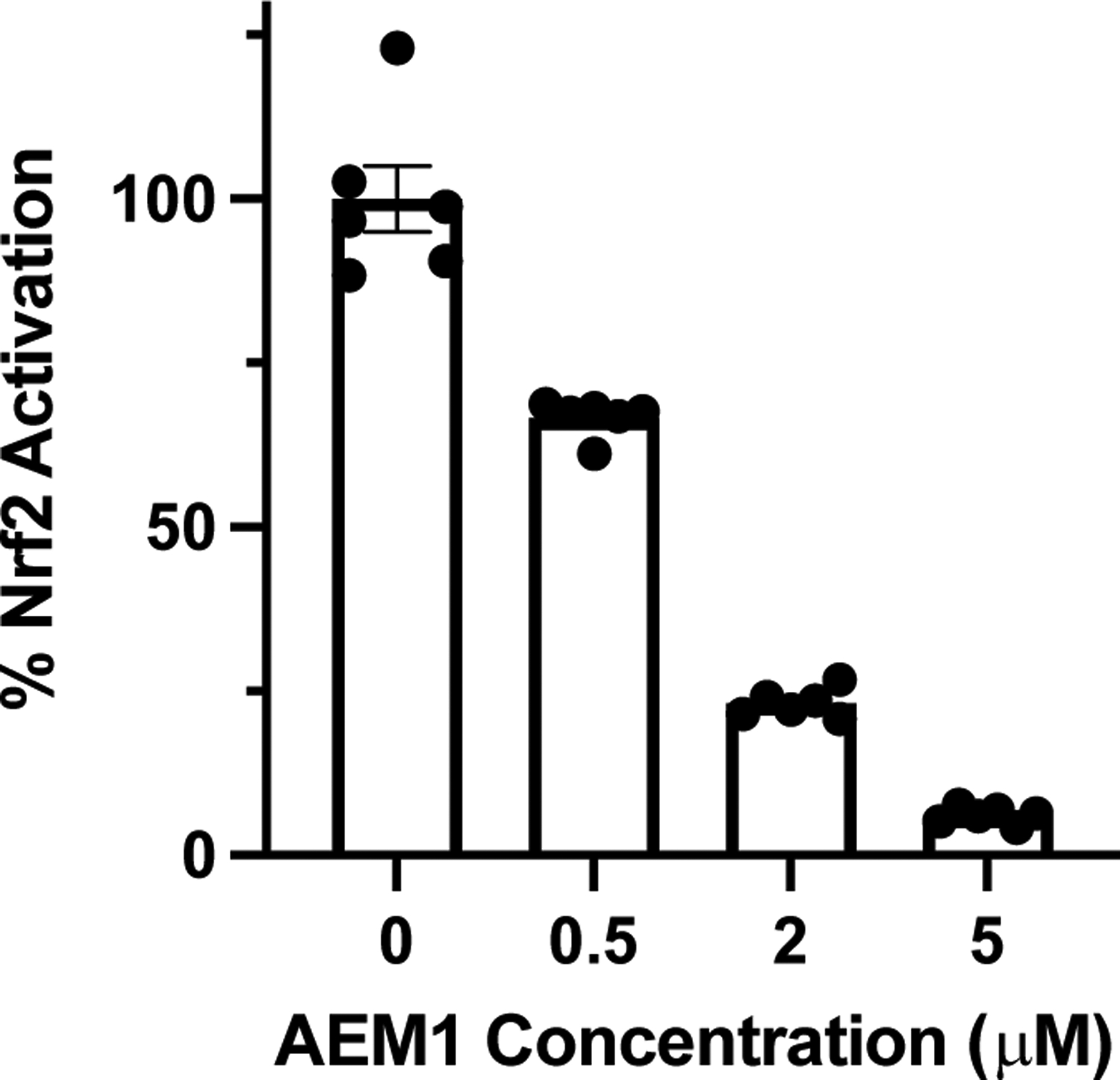
The Nrf2-inhibitor AEM1 dose-dependently blocked Nrf2 activation (p<0.05) induced in HepG2-ARE cells by treatment with PB123 (10 μg/mL).

First PB123 was determined to be nontoxic to HepG2 cells in the 0–50 μg/mL range, which exceeded the concentrations used in the rest of the study (Figure 1A), by measuring cell viability. Using the HepG2 cell line stably transduced with an ARE-promoter/luciferase-reporter construct [68], we determined that PB123 activates Nrf2 in a dose-dependent manner (Figure 1B). As expected, addition of the Nrf2-inhibitor AEM1 (0.5–5 μM) dose-dependently attenuated the Nrf2 activation (p<0.05) by PB123 (10 μg/mL)(Figure 2).

#### 3.1.2 Synergy

**Figure 3:**
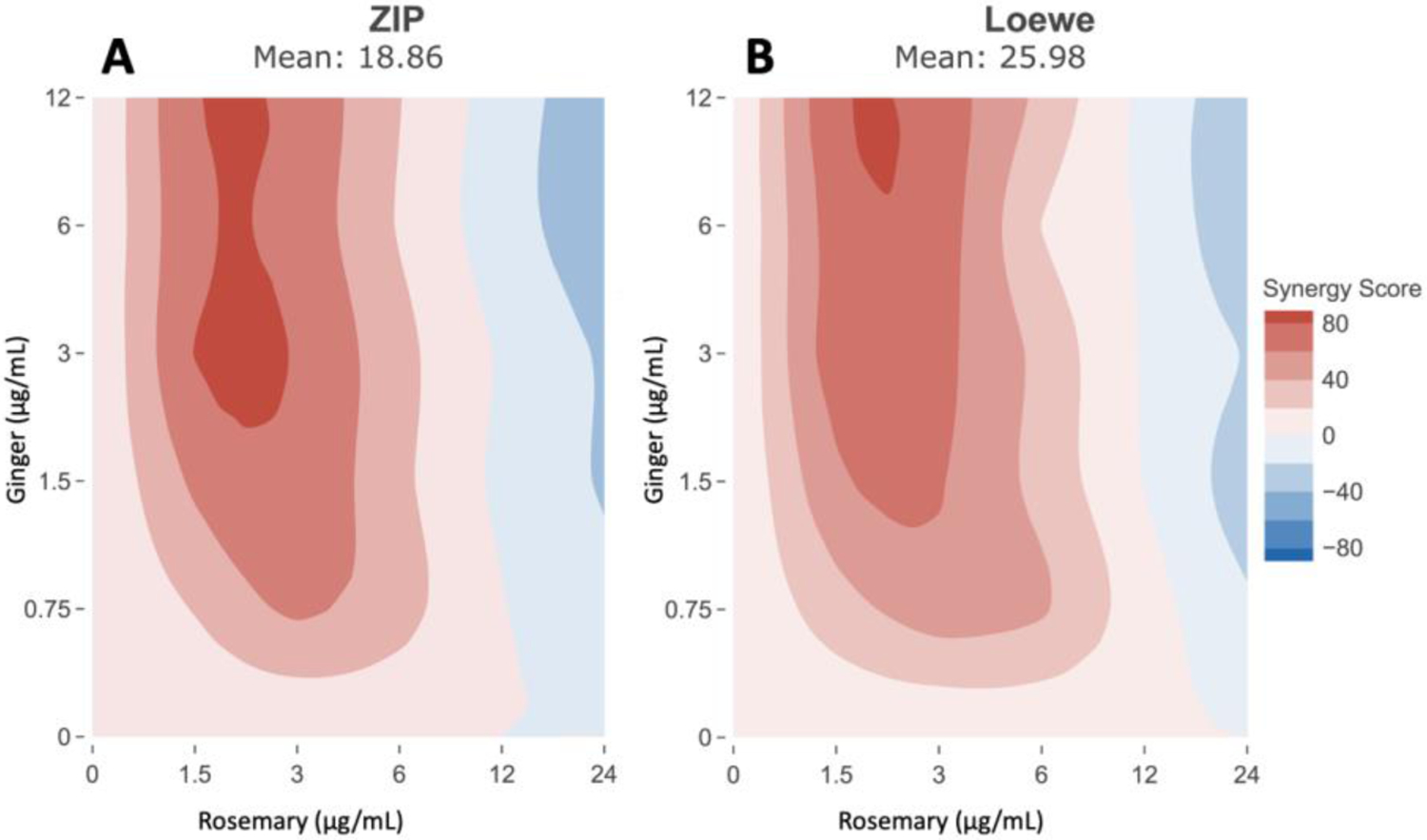
Combinatorial synergy analysis of HepG2 cells treated with Rosemary and Ginger extracts. Nrf2 activation by checkerboard combinations of Rosemary and Ginger extracts showed a strongly synergistic response as calculated using both (A) the Zero Interaction Potency and (B) the Loewe additive effect reference synergy models (synergyfinder.org).

Using the HepG2 cell line stably transduced with the ARE-promoter/luciferase-reporter [68], we found that the rosemary, ginger, and luteolin in PB123 combine synergistically for Nrf2 transcription factor pathway activation, with the most notable synergy observed between combinations of rosemary and ginger extracts. To model and visualize the synergy observed with rosemary and ginger for Nrf2 activation, we measured Nrf2 activation in HepG2-ARE cells with checkerboard combinations of rosemary and ginger extract concentrations and then used the Zero Interaction Potency (ZIP) and Loewe synergy. Both the (A) ZIP and (B) Loewe additive effect reference synergy models showed strongly positive synergy scores (synergyfinder.org) (Figure 3).

### 3.2 Gene Expression and Effects

#### 3.2.1 HepG2 Gene Expression by mRNA-seq Analysis

**Figure 4:**
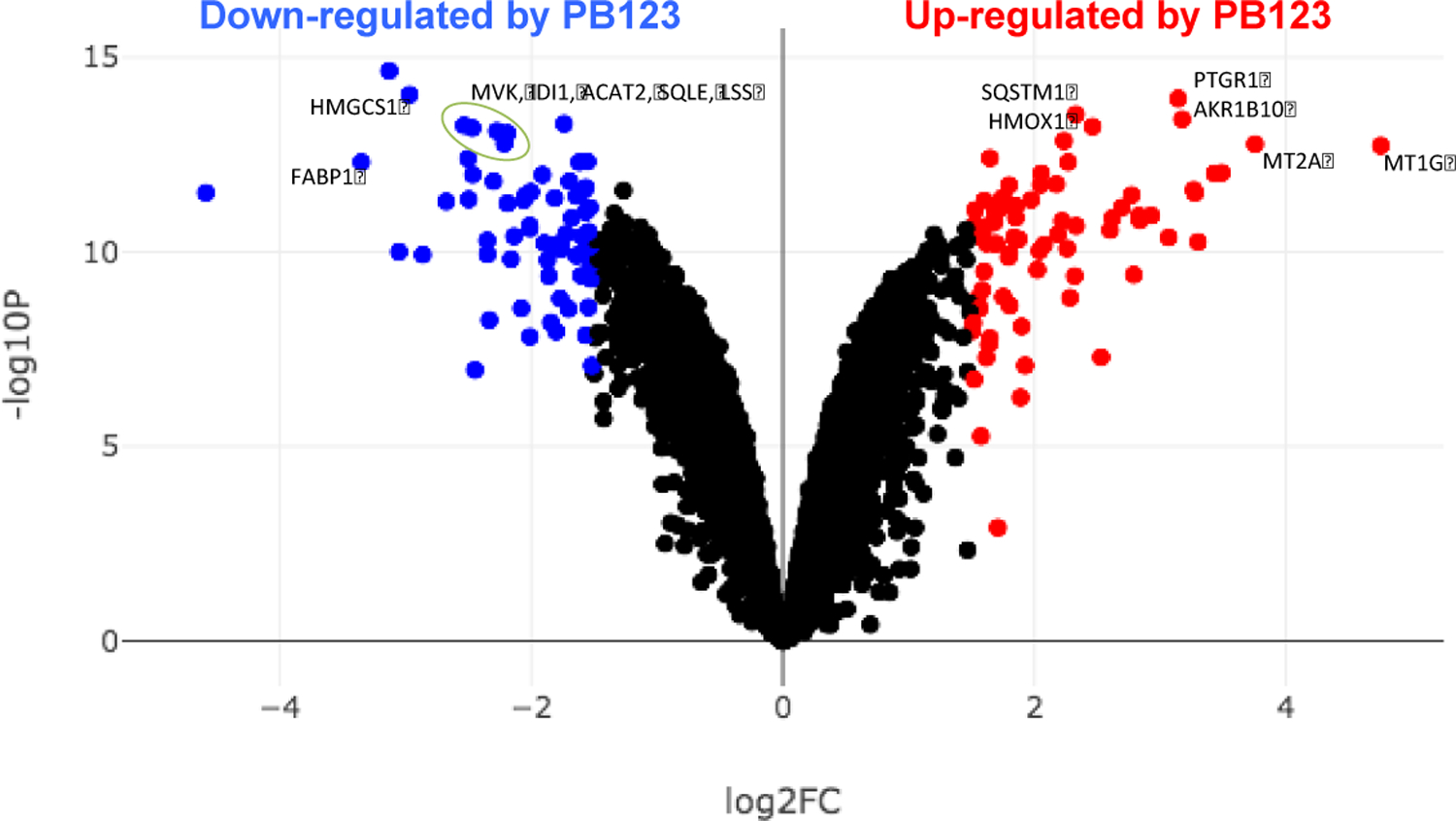
BioJupies was used to generate a Volcano plot of differentially expressed genes by HepG2 cells treated with PB123 12 μg/ml compared with untreated control HepG2 cells. Red indicates upregulated genes and blue indicates downregulated genes. Genes were selected with 2-fold change threshold. Gene symbols on the plots are used to mark some of the most significant genes changed, showing lipid and cholesterol synthesis genes downregulated and Nrf2-dependent genes upregulated.

In the present study the expression levels of 170 genes were increased and the expression levels of 247 genes were decreased by >2-fold in HepG2 cells treated for 16h with 12 μg/mL PB123, determined by mRNA-seq on cell treatment groups with 4 biological replicate samples per group (Figure 4).

**Figure 5:**
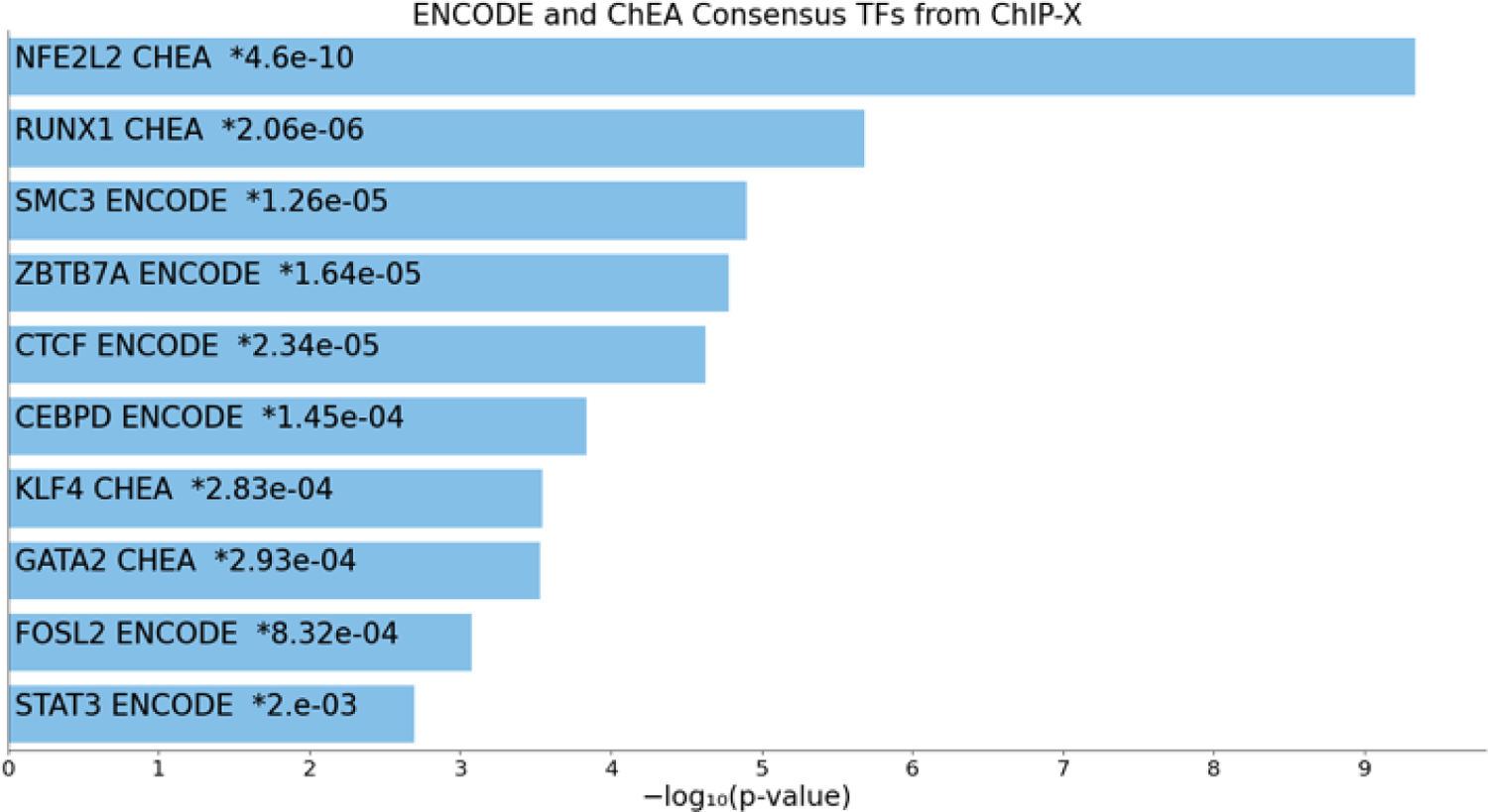
BioJupies was used to query ENCODE and ChEA consensus transcription factors with the DEG from the 12 μg/mL PB123 treatment mRNA-seq dataset and revealed prominent dependence on the Nrf2 (NFE2L2) transcription factor.

To quantify gene expression changes caused by PB123, we utilized the mRNA-seq approach to measure gene expression levels using separately cultured HepG2 cells. Evaluation of the dataset using BioJupies showed that the primary transcription factor affected was Nrf2 (NFE2L2) (Figure 5).

**Figure 6:**
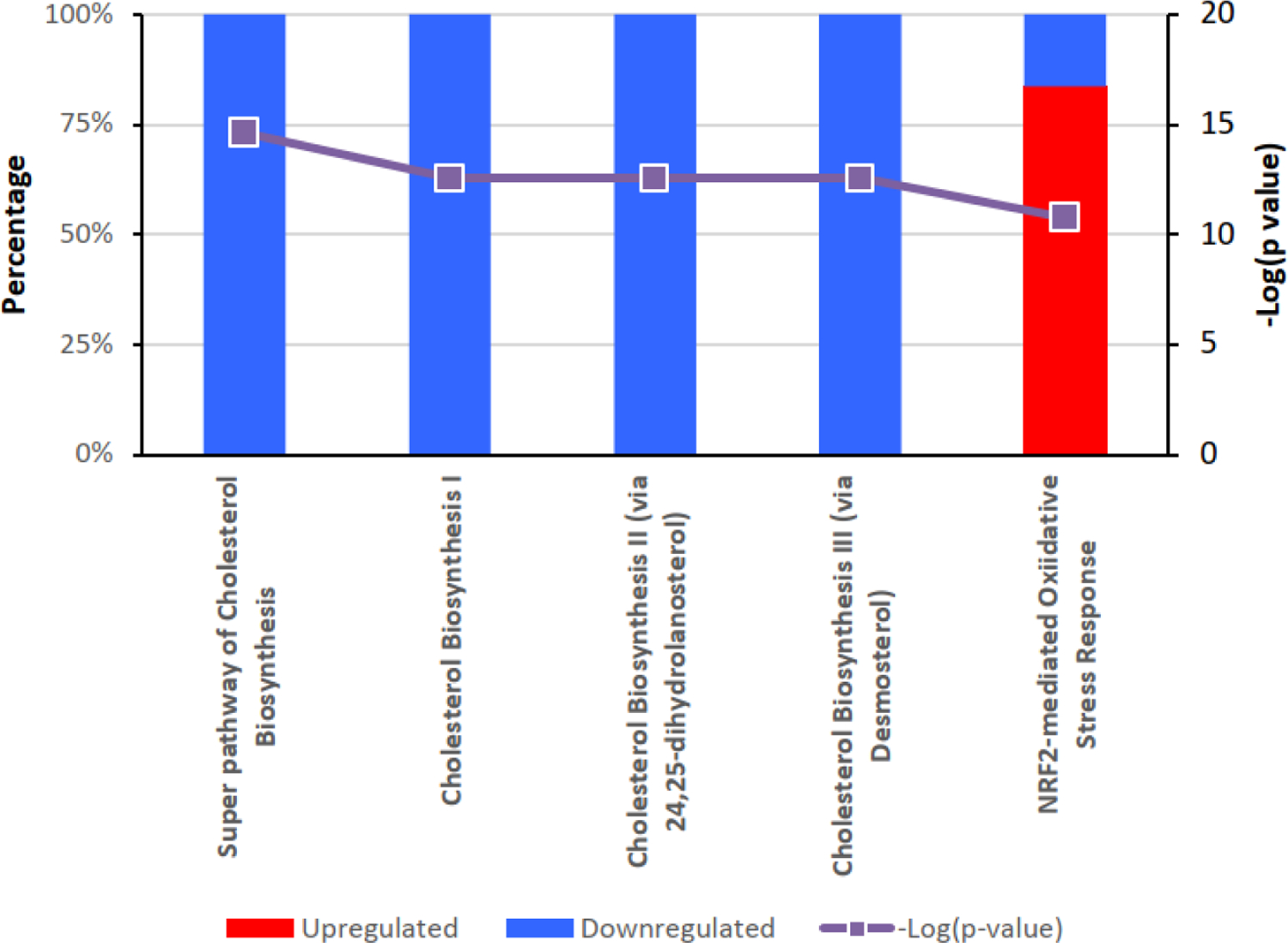
Ingenuity Pathway Analysis (IPA) indicates that PB123 downregulates genes in the cholesterol biosynthesis pathway and upregulates genes in the Nrf2 transcription factor pathway.

Ingenuity Pathway Analysis (IPA) likewise clearly demonstrated the primary importance of the Nrf2 transcription factor pathway in the differential gene expression induced by treatment of HepG2 cells with PB123 (Figure 6).

#### 3.2.2 Pathways Involved

**Figure 7:**
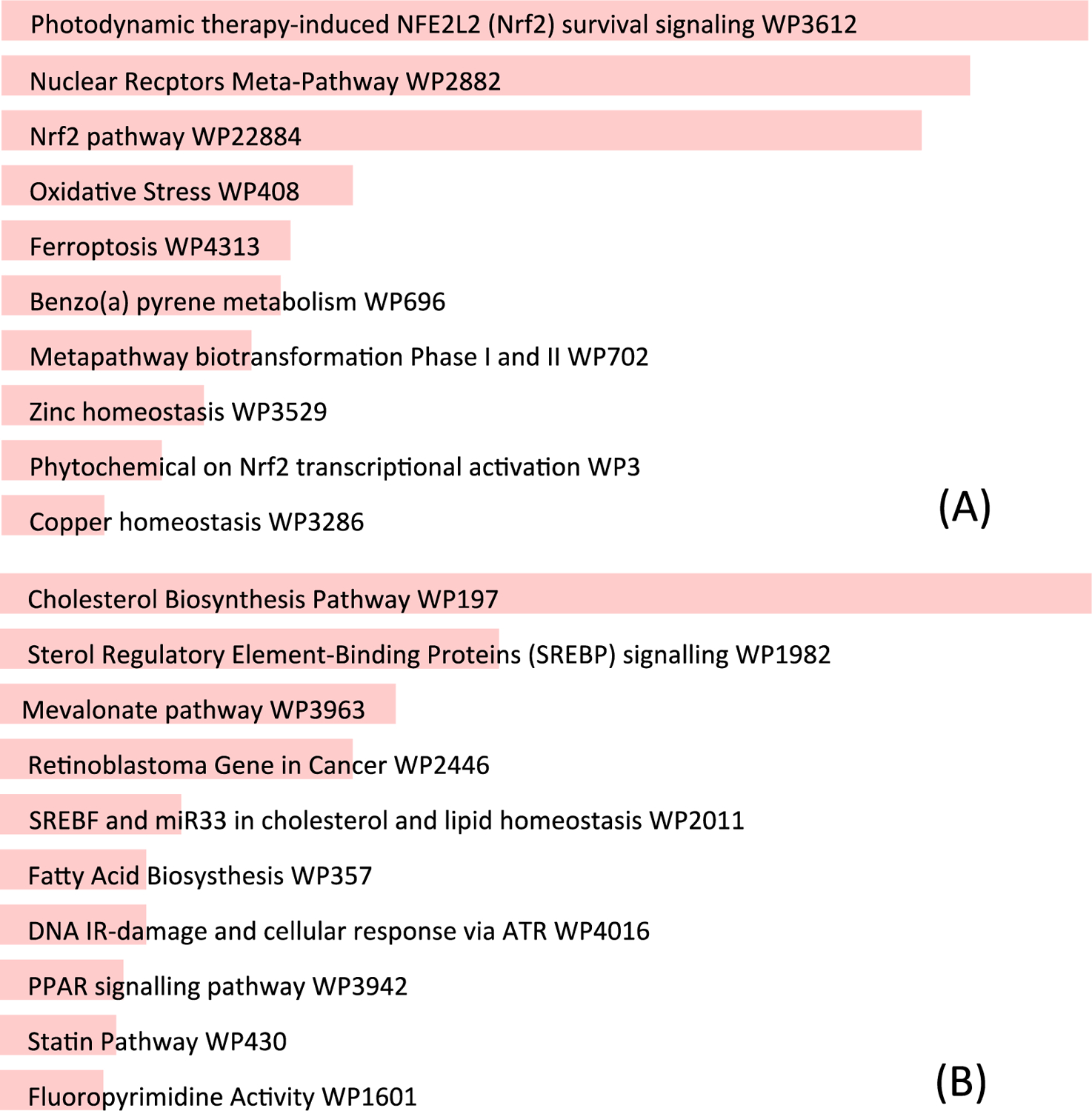
BioJupies was used to query Wikipathways to reveal the top pathways affected using the genes upregulated by PB123 (A) and downregulated by PB123 (B).

PB123 induced Nrf2 activation and upregulation Nrf2-dependent genes, and BioJupies and Ingenuity Pathway Analysis (IPA) identified the key involvement of the Nrf2 pathway for gene expression changes induced by PB123. Analysis of differentially expressed genes by IPA revealed that that PB123 upregulates genes in the Nrf2 transcription factor pathway and downregulates genes in the cholesterol biosynthesis pathway (Figure 6). Likewise, data analysis using BioJupies of upregulated and downregulated differentially expressed genes in the PB123-treated HepG2 cells indicated that the top affected pathways by Wikipathways analysis included NRF2 pathway (up) and Cholesterol biosynthesis pathway (down). The upregulated and downregulated Wikipathways results are shown in Figure 7.

#### 3.2.3 Cellular Lipids

**Figure 8:**
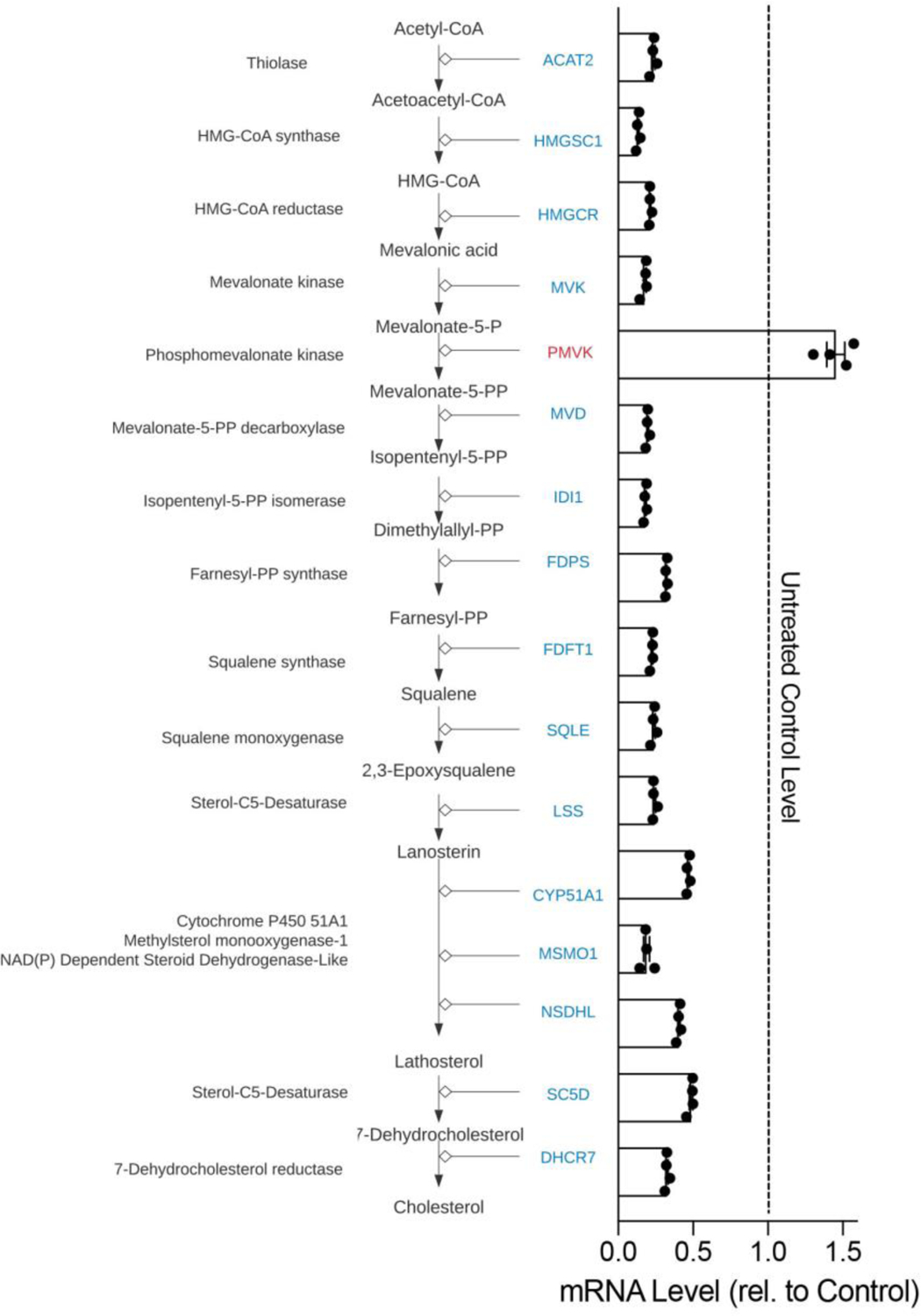
Cholesterol Biosynthesis Pathway genes from WikiPathways (WP197, https://www.wikipathways.org/index.php/Pathway:WP197). In our mRNA-seq dataset we found that all of the genes were significantly downregulated (shown in blue) by 12 μg/mL PB123 in HepG2 cells except *PMVK* (shown in red) compared to control-treated HepG2 cells (p<0.05, n = 4 per group). HMG-CoA reductase (*HMGCR*) is the rate-limiting enzyme in the pathway.

**Figure 9:**
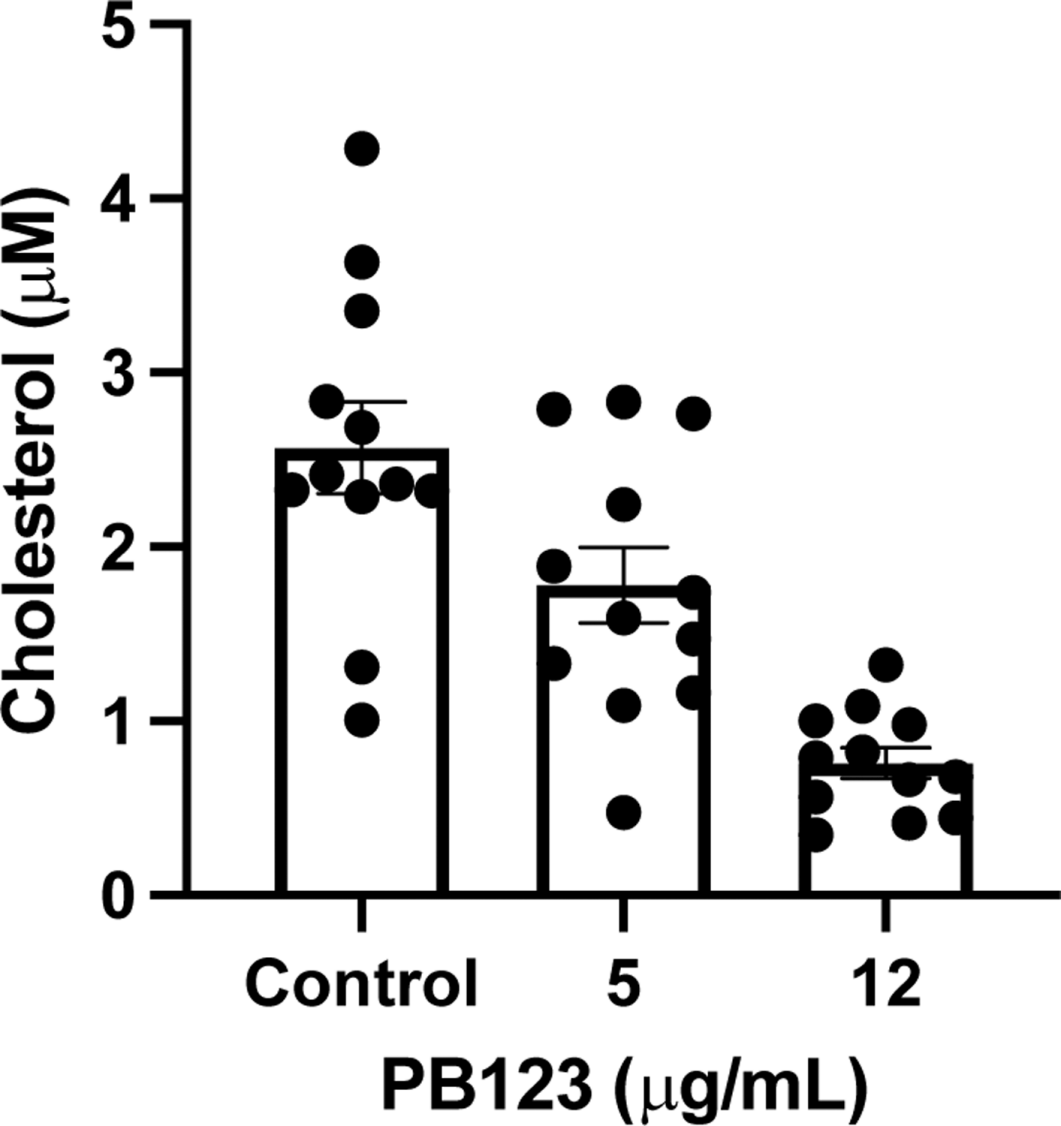
HepG2 total cholesterol. Total cholesterol levels were significantly reduced in HepG2 cells by 24 treatment with 5 or 12 μg/mL PB123 compared to untreated control HepG2 cells (p<0.05, n = 12 per group).

PB123 induced downregulation of the Cholesterol Biosynthesis Pathway, so we evaluated the individual genes involved in the Wikipathway gene set for WP197 by their mRNA-seq gene expression values with 4 biological replicates per group (the WP197 pathway and the mRNA expression data are shown together in Figure 8). Because the PB123-induced downregulation of the genes in the Cholesterol Biosynthesis Pathway was so extensive and consistent, we followed up with examination of PB123-induced changes in the cellular total cholesterol levels. Treatment (24h) of HepG2 cells with PB123 at 5 μg/mL and at 12 μg/mL significantly decreased intracellular total cholesterol levels in the cells (Figure 9). In related work, Li, et al., reported that total cholesterol in HepG2 cells was decreased by treatment with 6-gingerol which is one of the most active phytochemicals from ginger [77].

**Figure 10:**
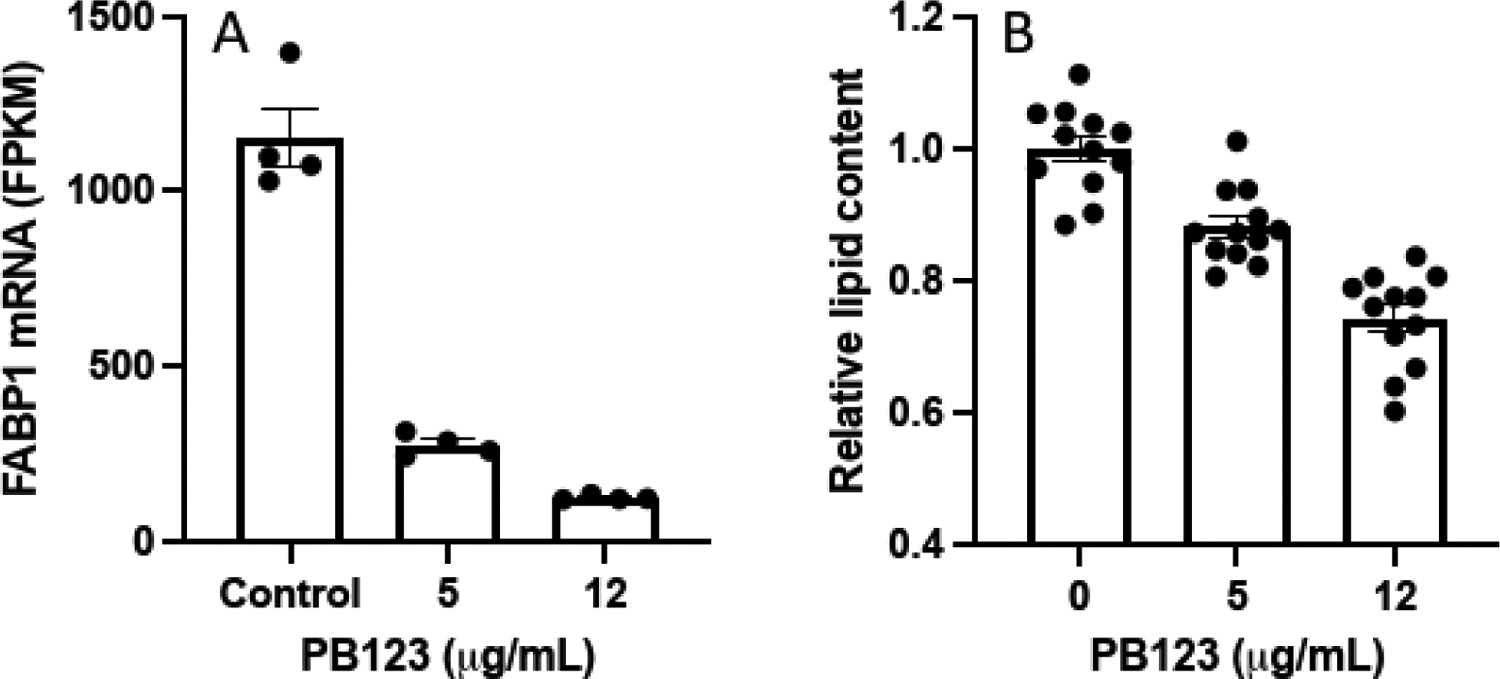
HepG2 intracellular lipids. We found that (A) *FABP1* was significantly downregulated by both 5 and 12 μg/mL PB123 in HepG2 cells compared to untreated control HepG2 cells (p<0.05, n = 4 per group), and (B) that intracellular lipid content was likewise significantly reduced by both 5 and 12 μg/mL PB123 in HepG2 cells compared to untreated control HepG2 cells (p<0.05, n = 12 per group).

Examination of the highly downregulated PPAR signaling pathway WP3942 (Figure 7B) along with highly downregulated genes in the volcano plot (Figure 4) indicated a possible role for PB123-induced downregulation of the fatty acid binding protein 1 gene *FABP1*. To follow up we evaluated the intracellular lipid droplet levels in HepG2 cells after 24h of treatment with PB123 (0, 5, and 12 μg/mL). The *FABP1* gene was significantly downregulated by both 5 and 12 μg/mL PB123 in HepG2 cells compared to control HepG2 cells (Figure 10A), and the HepG2 intracellular lipid content was significantly reduced by both 5 and 12 μg/mL PB123 (Figure 10B).

#### 3.2.4 HMOX1 mRNA and Protein

**Figure 11:**
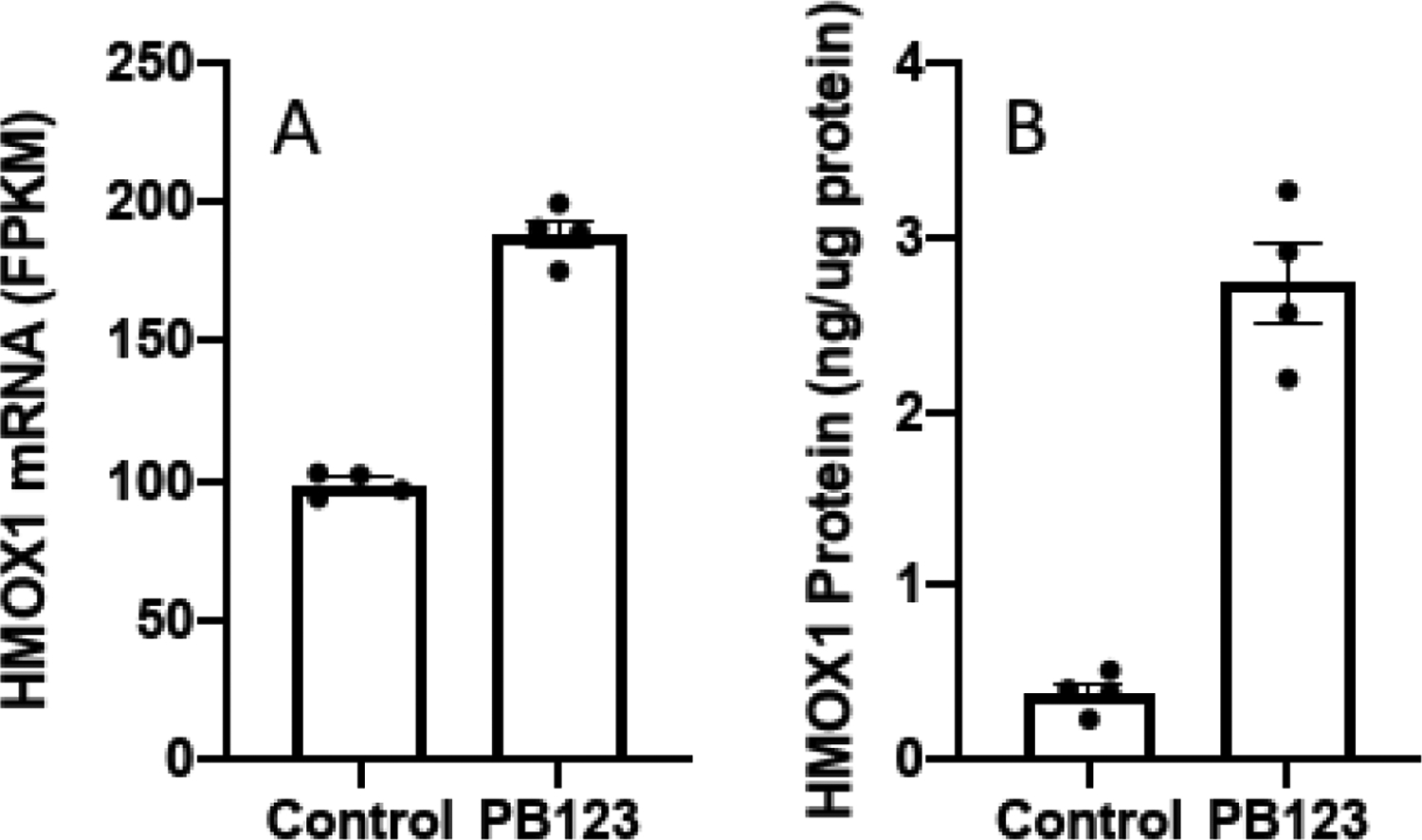
Heme oxygenase-1 gene expression and protein were increased in HepG2 cells by PB123 treatment. Treatment of HepG2 cells with PB123 (5 μg/mL, 24h) increased the both the levels of *HMOX1* gene expression determined by mRNA-seq (A) and the levels of HMOX1 protein determined by ELISA (B), as expected based on the large PB123-induced increase in Nrf2 activation (p<0.05, n = 4 in each case).

PB123 treatment of HepG2 cells (24h) increased the expression of the *HMOX1* gene (Figure 11A). As anticipated from the PB123-induced increase of *HMOX1* gene expression, levels of intracellular HMOX1 protein were also elevated by treatment of HepG2 cells for 16h with 5 μg/mL PB123 (Figure 11B).

#### 3.2.5 Oxidative Stress Protection

To assess cellular antioxidant defenses, we utilized the oxidant cumene hydroperoxide (CHP) to challenge HepG2 cells with an oxidative stress, with or without Nrf2 activation pretreatment with PB123. In a separate experiment, ERK1/2 kinase inhibition with PD98059, a selective and cell permeable inhibitor of the MEK/ERK pathway (10 μM PD98059, 30 min prior to treatment with PB123 or its individual components) was shown to decrease relative Nrf2 activation by PB123 and by each of the rosemary, ginger, and luteolin components (Table 1).

**Figure 12:**
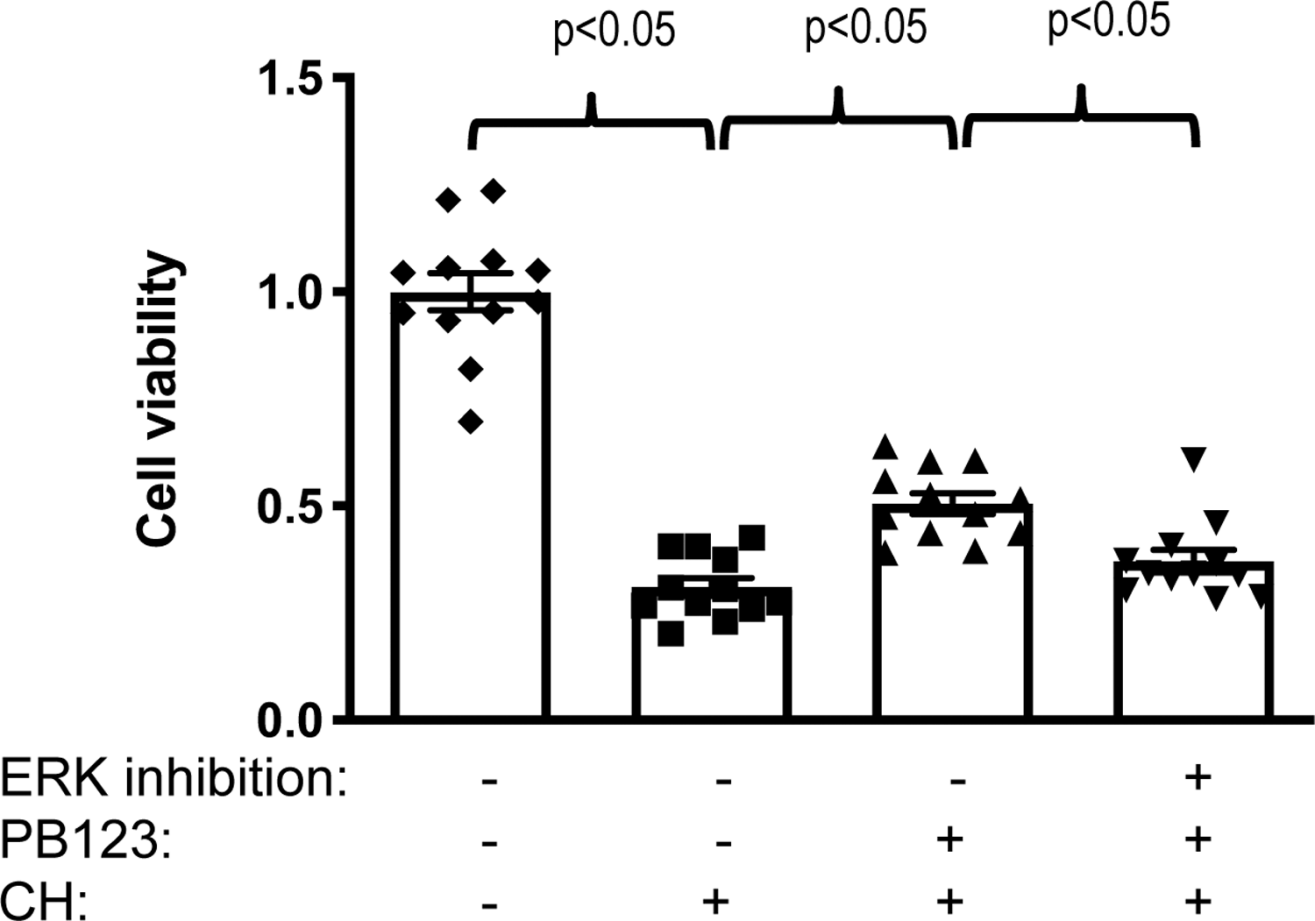
PB123 prevented loss of cell viability following challenge with an oxidative stress. Cytotoxicity was not observed (cell proliferation measured by CCK8 assay) in HepG2 cells treated for 16h with 5 μg/mL PB123 compared to untreated control cells. In HepG2 cells treated with 5 μg/mL PB123 or vehicle control for 18h, and then challenged with 25 μM cumene hydroperoxide (CH) or untreated control for 6 h, loss of cell viability (toxicity) was caused by CH challenge but this toxicity was partially attenuated (*p* < 0.05) by PB123 pretreatment. The protective effect of PB123 was blocked (p<0.05) by ERK1/2 kinase inhibition (10 μM PD98059, 30 min prior to PB123 treatment).

Based on that apparent ERK1/2 dependence of the Nrf2 response, the HepG2 cells were next cultured with 5 μg/mL PB123 for 16h, with or without 10 μM PD98059, added 30 min prior to the PB123, then the media was removed and the cells were washed with PBS prior to adding fresh culture media to prevent possible direct scavenging of applied oxidants by PB123 components. Next the cells were challenged with cumene hydroperoxide for 6 h and cell injury assayed by measuring cell viability. Pretreatment with 5 μg/mL PB123 protected against oxidative-stress-induced loss of viability in HepG2 cells that were subsequently challenged with 25 μM cumene hydroperoxide (Figure 12), but this protection was blocked if the ERK1/2 kinase was inhibited in the cells with PD98059.

## 4. Discussion

Aging has been associated with decreased ability to respond to stress-induced changes in gene expression in both animals [78] and humans [27]. Another key risk factor that increases with aging is dyslipidemia, which plays a key role in age-related cardiovascular disease [79]. Due to its well-documented role in the regulation of antioxidant and anti-inflammatory defense mechanisms, Nrf2 activation may play a key role in protection against age-related physiological decline. In the present work we show that the PB123 phytochemical dietary supplement combination based on rosemary extract, ginger extract, and luteolin synergistically activates the Nrf2 pathway (Figure 1), with especially strong synergy between the rosemary and ginger extracts (Figure 3).

The logic behind creating Nrf2 activators consisting of a combination of ingredients is threefold. First, the complexity of the Nrf2 activation/deactivation pathway is unusually great with dozens of control points having been demonstrated, ranging from promoter methylation and histone acetylation that regulate transcription of both *KEAP1* and *NFE2L2* (encoding Nrf2) genes, to numerous miRNAs that regulate translation of the transcripts [80–83], and luteolin participates at this epigenetic level of control. Once translated, regulation of the two proteins involves the more familiar array of covalent modifications involving electrophilic attack on the sulfhydryl groups of Keap1 which permit its release of Nrf2 [84]. Carnosic acid and carnosol from rosemary excel at this level of control as they behave as *pro-drugs* that possess little electrophilicity themselves but are converted to an electrophilic compound by the oxidative conditions at the site of the pathology that they are intended to alleviate [35, 85]. By contrast, dimethyl fumarate, an FDA approved Nrf2 activator used to treat relapsing multiple sclerosis [86], displays strong electrophilic toxicity and alkylating ability [35], much like the classical Nrf2 activator sulforaphane [87]. Their toxicity manifests due to non-specific reactivity with cellular protein thiols and glutathione depletion. Nrf2 regulation also results from a variety of covalent modifications to the Nrf2 protein which include phosphorylation by kinases such as PKC [88], PI3K [89], and MAPK [90], plus sumoylation [91] and ubiquitinylation [92], all of which modify Nrf2’s stability and ability to translocate to the nucleus. Once in the nucleus numerous other events regulate the ability of Nrf2 to regulate ARE/EpRE driven genes. These include acetylation/deacetylation of Nrf2 by Creb binding-protein and Sirt1 [93]. Acetylated Nrf2 was found to have augmented binding to the ARE promoter establishing acetylation as another regulatory mechanism for Nrf2 [94]. BACH1 is a negative regulator of Nrf2 and silencing it with siRNA increased expression of Nrf2 regulated genes [95]. Furthermore, this work suggested an interesting age-dependent difference in the expression profile of Nrf2-regulated genes due to increased Bach1 protein in older individuals. What appears to be another major controller of Nrf2 activity is the mechanism that ejects it from the nucleus, terminating its activity and tagging it for proteasomal degradation. The process is driven by activation and nuclear translocation of cytosolic Fyn kinase upon phosphorylation by GSK3β. The active Fyn in the nucleus tags Nrf2 for ejection, ubiquitination, and protesomal destruction. Interestingly, the process is facilitated by and central to acetaminophen toxicity [96]. Luteolin and carnosic acid were found to inhibit the ejection of Nrf2 as follows: luteolin and carnosic acid both activate PI3K [89, 97] which not only phosphorylates Nrf2 to facilitate its translocation to the nucleus but which also activates Akt; Akt phosphorylates GSK3β, *inactivating* it, which prevents it from activating cytosolic Fyn; the inactive Fyn remains in the cytosol and cannot enter the nucleus to contribute to the expulsion of Nrf2, meaning that active Nrf2 will have a longer duration in the nucleus, increasing its efficiency.

The second advantage of using multiple compounds to activate Nrf2 is that synergy that may result. Clearly, the activation of Nrf2 is not a linear pathway with a single rate-controlling step. Many of the contributing control points mentioned above may require the cooperation of other control points to realize maximal effect. For example, release of Nrf2 from Keap1 is necessary but not sufficient for a maximal effect—Nrf2’s journey requires covalent modification by several possible mechanisms as mentioned above for nuclear entry. We have demonstrated substantial amounts of synergy by combining ingredients with diverse contributions to the process of Nrf2 activation [42, 67] as in the present study.

The third logical rationale for using a combination of ingredients to activate Nrf2 lies in the appreciation that activation is the result of not only a large number of control points, some positive and some negative, but that with a Nrf2 activation *network* that’s this complicated (as opposed to a simple, linear *pathway* of activation), one may certainly need to combine agents that act at multiple key points in the network, with combined minimal toxicity, to expect a useful end result. As an example, single compounds that act only to release Nrf2 from Keap1 may indeed result in a rapid influx of Nrf2 to the nucleus, but it will be followed by an increased expulsion of that Nrf2 from the nucleus unless the Fyn-dependent export is simultaneously inhibited. Without slowing the exit pathway, Nrf2 stores in the cytosol would quickly become depleted, especially in elderly individuals as the rate of Nrf2 synthesis slows with aging [78, 98]. The outdated paradigm of “One disease, one drug” that has driven recent decades of pharmacological thinking does not apply to network pharmacology [99].

In the present work, examining the upregulation of a well-established Nrf2-dependent gene to support PB123-induced Nrf2 activation, we found that the levels of the Nrf2-dependent *HMOX1* gene (Figure 11A) and HMOX1 protein (Figure 11B) were increased in HepG2 cells by PB123 treatment. In addition, PB123 and its individual components showed ERK1/2 dependence for Nrf2 activation (Table 1) as well as for PB123-induced protection against cumene hydroperoxide-induced oxidative stress and loss of cell viability in HepG2 cells (Figure 12).

The PB123-induced downregulation of genes in the Cholesterol Biosynthesis Pathway (see Figure 8) is notable. PB123 treatment decreased the expression of fifteen genes involved in cholesterol biosynthesis (*ACAT2*, *HMGSC1*, *HMGCR*, *MVK*, *MVD*, *IDI1*, *FDPS*, *FDFT1*, *SQLE*, *LSS*, *CYP51A1*, *MSMO1*, *NSDHL*, *SC5D*, and *DHCR7*) and increased the expression of one gene in the pathway (*PMVK*). This result suggest that biosynthesis of cholesterol in HepG2 cells might be decreased by treatment with PB123, which was supported in a follow-up experiment demonstrating decreased levels of total cholesterol in HepG2 cells cultured with PB123 (Figure 9). Dietary approaches may be useful to help with healthy aging, with the goal of attenuating age-related dyslipidemia in combination with attenuating age-related oxidative stress and inflammation.

FABP1, also known as liver fatty acid binding protein, has high affinity for fatty acids by means of two fatty acid-binding sites [100]. FABP1 plays an important role in fatty acid uptake in HepG2 cells [101], leading to hypothesis that FABP1 may contribute to liver steatosis. Notably, silencing *FABP1* decreased hepatic steatosis, inflammation, and oxidative stress in a mouse model of nonalcoholic fatty liver disease (NAFLD) [102]. Further, exercise was found to decrease *FABP1* gene expression in mice and protect against NAFLD [103], and in human subjects urinary FABP1 protein levels were lowest in the fittest subjects, those with higher levels of muscle strength and aerobic fitness [104]. Recently, increased FABP1 levels in human subjects has been identified as a biomarker in diabetic nephropathy [105]. In our HepG2 cell experiments, the pronounced downregulation of *FABP1* by PB123 (Figure 10A) was accompanied by a significant decrease in intracellular fatty acid levels in the cells (Figure 10B), similar to the results previously reported in HepG2 cells with *FABP1* knocked down using antisense RNA [101].

Extensive prior work supports beneficial effects of the individual components of PB123 (rosemary, ginger, and luteolin) for normalizing lipid and cholesterol levels [106–114]. In our current work those benefits are likewise noted, along with the activation of the Nrf2 transcription factor, beneficial effects on gene regulation, and protection against oxidative stress.

## 5. Conclusions

We found that the PB123 phytochemical combination is a potent Nrf2 activator with significant synergy between the rosemary and ginger components. Pathway analyses of genes differentially expressed by PB123 treatment of HepG2 cells in mRNA-seq experiments revealed prominent upregulation of genes in the Nrf2 pathway and downregulation of genes in the cholesterol biosynthesis pathway. Further, pretreatment with PB123 protected cultured HepG2 cells against an oxidative stress challenge caused by exposure to the organic oxidant cumene hydroperoxide. In addition, the downregulation of lipid uptake and cholesterol biosynthesis genes were accompanied by decreased intracellular lipid levels and decreased total cholesterol levels in PB123-treated HepG2 cells. The Nrf2 activation, highly synergistic effects between the rosemary and ginger ingredients, differential gene expression in pathways that pertain to increased cytoprotection, decreased cholesterol synthesis, and decreased intracellular lipid accumulation by the dietary supplement PB123 support its use to promote healthy aging.

## Figures and Tables

**Table 1:** Decrease of Nrf2 activation with ERK1/2 inhibition.

	Nrf2 Activator Alone	With ERK1/2 Inhibitor
Rosemary	100%	51 ± 4%
Ginger	100%	58 ± 8%
Luteolin	100%	42 ± 12%
PB123	100%	58 ± 4%
